# The Effect of Squad Rotation on Physical Activity at the 2018 World Cup in Russia. Analysis the Most Exploited Players of the 4 Best Teams

**DOI:** 10.3389/fpsyg.2021.726207

**Published:** 2021-09-17

**Authors:** Michał Kołodziejczyk, Paweł Chmura, Marek Konefał, Jan Chmura, Andrzej Rokita, Marcin Andrzejewski

**Affiliations:** ^1^Department of Team Games, University School of Physical Education, Wrocław, Poland; ^2^Department of Biological and Motor Sport Bases, University School of Physical Education, Wrocław, Poland; ^3^Department of Methodology of Recreation, Poznań University of Physical Education, Poznań, Poland

**Keywords:** soccer, match analysis, rotation of players, high intensity, distance covered, sprints

## Abstract

The purpose of this study was to examine how the four best teams in the 2018 Football Men's World Cup rotate by squad and how this impact the physical activity of the teams in consecutive rounds. The study sample consisted of the 31 players of the 4 best teams, who played in every tournament match, except for the third game of the group stage. The analysis included 186 observations and was carried out on the most exploited players (MEP) excluding goalkeepers, who played at least 450 mins (5 full matches) in the tournament. The analysis was conducted using data collected by an advanced motion analysis system known as STATS®. The selected physical activity parameters analyzed included: total distance covered (m/min), distance covered at various intensity ranges (m/min), top speed (km/h), and number of sprints performed. It was found that all four teams in the third match of the group stage have performed the largest number of rotations with most exploited players and introduced the highest number of rested players (7.75 ± 2.06). A significant increase was observed between the second and fourth match in the 0–7 km/h distance covered (37.99 ± 3.19–39.23 ± 3.35 m/min) and the top speed (28.12 ± 2.22–29.21 ± 2.64 km/h)—*p* < 0.05. Furthermore, MEPs in the knockout stage, used pacing strategies by increasing the amount of low-intensity running to maintain high intensity during the game. From a practical point of view, this investigation shows that squad rotation can be a valuable support to a pacing strategy by players.

## Introduction

Analyzing transitioning from club to national teams, found that international footballers are exposed to an increase in relative load during the transition from club-to-camp. Pre-World Cup training camps functions to ensure that the players are tactically, physically and mentally prepared for tournament demands (Noor et al., 2019). However, complicating this pretournament planning is the need to appropriately balance training load and recovery within a truncated period between home club commitments and the tournament (Buchheit and Dupont, 2018). This is due to the problematic pretournament period, which creates difficulties of quantifying the load in international footballers (Buchheit, 2017). Given the high number of matches played in modern soccer, a crucial question for the “productivity” of players and the optimal organization of national leagues and international championships is whether players are allowed the necessary rest between consecutive matches (Scoppa, 2013). Indeed, no evidence exists to show that match performance is actually affected in players not fully recovered, with possibly a greater case for use in injury prevention schemes. Collecting data is problematic due to staff and player buy-in and compliance as well as the logistical burden and limitations of monitoring tools and protocols (Carling et al., 2018). Therefore, one of the factors is investigation of the squad rotation during international tournament.

Squad rotation and player availability (particularly remaining injury free) are key issues in coping with the high demands of contemporary training and match-play (Strudwick, 2013). There are many reasons to make rotation of players, understood as replacing and introducing players in the starting 11 in consecutive matches, it is modifying tactical behavior (Hirotsu and Wright, 2002), preventing fatigue (Bradley et al., 2014), replacing yellow card players (Ascari and Gagnepain, 2006) and replacing injured or underperforming players (Hills et al., 2018). According to James et al. (2002), coaches can manipulate the squad and potentially gain a competitive edge, the opposition by strategically rotating players on and off the field. Varley et al. (2018) highlighted the use of rotation after the team qualified for the knockout stage after winning the first two games in the group stage. In this situation, the coaches, who are sure of promotion in the tournament, let the most important players rest. Furthermore, in 2018 Palluci et al. analyzed the Brazilian league, they concluded that the rotation of players certainly contributed to the better results. From a pragmatic point of view, it is important to the analysis of managing the most exploited players and squad rotation effect on physical activity in consecutive matches.

Contemporary international football tournaments are characterized by a high number of matches condensed into a short period of time (Silva et al., 2017). This emphasizes the importance of rotation within teams during tournament matches (Carling et al., 2014; Bekris et al., 2020). Matches are held every few days, and each team plays seven matches in a month, which means that the players play for a total of 630 minutes, not excluding the extra time that may be added in the knockout stage (Król et al., 2017). The coaches make changes during tournaments such as World Cup to allow time for those with less exposure to the match and to prevent the accumulation of fatigue among the most exploited players (Hills et al., 2018). In addition, the investigation shows, that rotation was widely adopted to justify maintaining player performance during congested periods (Dupont et al., 2010; Carling et al., 2012; Djaoui et al., 2014; Dellal et al., 2015). According to Varley et al. (2018) also stated on average 3 players per team were more exploited than the others and played in 100% of the group stage matches (ranging from 1 to 5 players per team). Moreover, according to Carling et al. (2015), the squad rotation of club-level soccer shows that <40% of the squad will have to play full time in the next matches. In addition, the chance of rotation of resting players in consecutive matches is relatively low in both tournament and home competitions, affecting 30% of the teams in the tournament and remains significant for coaches (Varley et al., 2018). However, during tournaments the coaches may limit the rotation to the best and most exploited players. Therefore, despite the use of rotation, some players will be more exploited and play in most or all matches.

Understanding player pacing and its relationship to the introduction of appropriate player rotation can be a valuable tool for coaches in developing tactical strategies during busy schedules and making better decisions to improve team performance (Mujika et al., 2018). Observations from the 2010 World Cup in RPA and the 2014 World Cup in Brasil indicate a steady tendency to increase the intensity of match performance (Oh Sang et al., 2011; Chmura et al., 2017b). According to Chmura et al. (2017a) it is an indication of the high demands of players' speed and endurance abilities necessary for their motor activities in the key rounds of top-level soccer tournaments. Winning a top ranked soccer tournament requires players to cover longer mean total distances as well as longer distances at high intensity during a match. This implies that elite soccer players must possess high levels of aerobic and anaerobic capacity. Therefore, it is interesting to analyze the physical activity attained by soccer players in consecutive rounds during an international tournament. Moreover, it is also interesting that most exploited players are able to maintain particularly high activity in the key matches in the semi-finals and finals and the third place match. In addition, during top-level tournaments, players must be prepared for maximum motor skills despite the limited time available for rest.

Therefore, the main objective of the study was to analyse the squad rotation and their effect on physical activity. Hence, there were the most exploited players of the top 4 teams analyzed during the consecutive seven games of the tournament. The desired research outcome was to obtain the information on the recurring steps coaches had taken within their teams' squads and their effects on changes in selected physical activity parameters. It was assumed that the most exploited players, as a result of obtaining a few extra days without match effort, maintain or increase physical activity in the next tournament matches.

## Methods

### Players and Match Data

The study sample comprised 186 observations generated by 31 players (13 defenders, 11 midfielders, and 7 forwards, without goalkeeper) through 6 matches each during the 2018 Football Men's World Cup and the analysis used the results obtained up to 90 mins with extra time. Mean body height among the players was 183.26 ± 6.74 cm, mean body mass 78.39 ± 8.82 kg, and mean age 26.52 ± 3.12 years. The average break between matches was 4.58 ± 0.78 days. The teams won all consecutive matches to the semi-final stage, where they faced each other directly, except for France's draw against Denmark and England's loss against Belgium in the third match of the group stage. The weakest opponent in the FIFA ranking on which the analyzed 4 best teams played, was Russia −70th place, and other opponents had a higher place in the ranking before start of the World Cup. This study maintains the anonymity of the players following data protection law, which is conducted in compliance with the Declaration of Helsinki and was approved by the local Board of Ethics.

### Data Collection and Analyses

The physical activity of players was measured using an advanced motion analysis system known as STATS® (Chicago, IL, USA), operated at 25 frames per second and allowing for simultaneous tracking of players' actions during each second of the game, in all sections of the soccer pitch. The validity and reliability of this system for taking such measurements have been described in detail elsewhere (Ramos et al., 2017; Linke et al., 2018). Match data were retrieved from the official website of FIFA (FIFA, 2018) and have been previously used for FIFA World Cup related research by da Mota et al. in the review 2015, which discussed in detail the tracking and coding process of the FIFA official dataset.

To better understand the intensity of match activity in consecutive rounds of the tournament, the recorded variables including selected physical activities of the most exploited players have been converted to meters per minute: total distance covered, distance covered at intensity ranges of 0–7, 7–15, 15–20, 20–25 km/h, above 25 km/h and number of sprints performed. Top speed has been converted to kilometers per hour. Selected physical activities have been used in many earlier publications (Morgans et al., 2014; Chmura et al., 2017b; Liu et al., 2020; Kołodziejczyk et al., 2021).

### Statistical Analysis

Data are presented as means ± SD in the text and tables. All variables were checked to verify their conformity to a normal distribution (Shapiro–Wilk test). Repeat-measures ANOVA was applied in comparing mean values for these. Where a significant difference was found, a Fisher LSD *post hoc* test was performed to assess differences between means. The level of statistical significance was set at *p* ≤ 0.05. All statistical analyses were performed using the Statistica ver. 13.2 software package (from StatSoft. Inc., USA).

## Results

Table 1 presents information about squad rotation in consecutive matches of the top 4 teams at the 2018 World Cup in Russia. Analysis shows that most rotations were made in the third match of group stage (7.75 ± 2.06 rested players). In the other matches, the same number of rotations were performed (2.75 ± 2.75 rested players). Moreover, the table shows the average game time of all analyzed players. It shows that the most significant difference in means of this parameter occurred between 2 and 4 matches (88.83 ± 12.6 to 107.15 ± 18.52).

**Table 1:** Squad rotation in consecutive matches of the top 4 teams at the World Cup in Russia.

**Variables**	**Group stage**			**Knockout stage**				***F* (sig)**	**SSD (*p* ≤ 0.05)**
	**1st (1)**	**2nd (2)**	**3rd (3)**	**Round of 16 (4)**	**Quarter-final (5)**	**Semi-final (6)**	**Final/3rd place match (7)**		
Number of rotation (number)	2.75 ± 2.75	2.75 ± 2.75	The greatest number of rotations 7.75 ± 2.06	2.75 ± 2.75	2.75 ± 2.75	2.75 ± 2.75	2.75 ± 2.75	**50.00 (0.000)**	3>1,2,4,5,6,7
Total time (min)	89.36 ± 17.37	88.83 ± 12.6		107.15 ± 18.52	99.97 ± 17.68	110.5 ± 18.43	90.63 ± 13.41	**13.17 (0.000)**	2 <4 5 <6 6>7

*Bold indicates significant differences between rounds*.

Table 2 presents information about the physical activity of the most exploited players in the consecutive matches at the 2018 World Cup in Russia. Analysis of the average values between 2 and 4 matches during the tournament showed a significant increase in the distance covered with an intensity of 0–7 km/h (37.99 ± 3.19 to 39.23 ± 3.35) and total speed (28.12 ± 2.22 to 29.21 ± 2.64). Moreover, significant changes were noted between Semi-final and Final/3rd place match. Total distance covered decreased (from 106. 99 ± 10.23 to 99.06 ± 12.57), the distance covered with an intensity of 7–15 km/h decreased (from 41.13 ± 3.87 to 39.26 ± 4.93) and the distance covered with an intensity above 25 km/h increased (from 2.18 ± 1.25 to 2.56 ± 1.37).

**Table 2:** Physical activity parameters of four best teams in consecutive matches at the World Cup (included extra time), converted to meters per minute except number of sprints and top speed.

**Variables (m/min)**	**Group stage**			**Knockout stage**				***F* (sig)**	**SSD (*p* ≤ 0.05)**
	**1st (1)**	**2nd (2)**	**3rd (3)**	**Round of 16 (4)**	**Quarter-final (5)**	**Semi-final (6)**	**Final/3rd place match (7)**		
Distance covered (km)	106.75 ± 7.93	102.41 ± 10.44	The greatest number of rotations 7.75 ± 2.06	102.62 ± 11.07	103.47 ± 14.02	106.99^*^± 10.23	99.06^*^± 12.57	**3.51 (0.005)**	6>7
0–7 km/h distance covered	37.71 ± 3.6	37.99^*^± 3.19		39.23^*^± 3.35	39.06 ± 3.03	39.12 ± 3.13	39.84 ± 3.66	**6.03 (0.000)**	2 <4
7–15 km/h distance covered	41.52 ± 4.33	41.80 ± 4.08		40.91 ± 5.07	41.05 ± 4.22	41.13^*^± 3.87	39.26^*^± 4.93	**4.00 (0.002)**	6>7
15–20 km/h distance covered	14.03 ± 4.61	13.34 ± 3.92		12.47 ± 3.66	13.19 ± 4.23	13.19 ± 4.16	12.99 ± 3.77	1.60 (0.164)	–
20–25 km/h distance covered	5.67 ± 1.87	5.35 ± 1.84		4.89 ± 1.44	5.19 ± 1.69	5.45 ± 1.87	5.44 ± 1.48	2.07 (0.073)	–
>25 km/h distance covered	2.07± 1.51	1.75 ± 1.05		2.02 ± 1.28	1.94 ± 0.92	2.18^*^± 1.25	2.56^*^± 1.37	**4.08 (0.002)**	6 <7
Sprints (number/min)	0.35 ± 0.12	0.32 ± 0.12		0.30± 0.10	0.32± 0.11	0.33 ± 0.12	0.34± 0.10	2.28 (0.059)	–
Top speed (km/h)	28.87 ± 2.69	28.12^*^± 2.22		29.21^*^± 2.64	29.14± 1.9	28.72 ± 2.32	28.87± 2.12	**1.28 (0.027)**	2 <4

*Bold indicates significant differences between rounds*.

* *Significance between rounds*.

## Discussion

The purpose of this study was to examine the squad rotation and their impact on physical activity in consecutive rounds of the tournament of the four best teams in the 2018 Football Men's World Cup. To our knowledge, this study is the first analysis of the most exploited players during a high-level tournament. They were obtained the information on the recurring steps coaches had taken within their teams' squads and their effect on changes in selected physical activity parameters. The main observation obtained from the analysis of the collected data is that the rotation was done in the third match of the group phase, and that the MEP in the following rounds ran more and more at low intensity to maintain the key high intensity and sprints.

According to Julian et al. (2021), insufficient recovery between successive matches and the occurrence of congested schedule periods has been a factor that affects performance. All studied MEP ended their season between May 12 (German league) and May 26 (Champions League). The analyzed teams played all pretournament friendly matches between 28 May (France) and 11 June (Belgium). Thus, the potential rest time of the analyzed players between the end of the season and the start of the national team camp could last only a few days. This is important because before the World Cup, players come to the training camp from different leagues, have different loads, some are overtrained, others are undertrained. The variation in the level of preparation of players for the tournament is so great that coaching staffs are not able to prepare all players for the tournament (Buchheit and Dupont, 2018; Noor et al., 2019). Due to winning the first two matches in the group stage, the coaches of the analyzed teams used the third game to extend the rest period and to better regenerate the MEP. This is confirmed by Varley et al. (2018), who noted that after the first two games of the group phase comes the best time for rotation. Moreover according to Carling et al. (2018) the present findings on the extent of the injury problem lend weight to effective squad rotation strategies. This can also be seen in our analysis, which shows that all 4 teams analyzed rotated an average of as much as 7.75 ± 2.06 players per 10 in the basic squad. Moreover, one of the reasons for the rotation in the third group stage match may have been to better prepare the MEP for the knockout phase. It should be noted that the teams' strategies and tactics are likely to be very different when playing to obtain points from playing for immediate promotion (Liu et al., 2015).

Soroka and Peñas (2016), analyzing players who played in every consecutive match of the group stage at the 2014 FIFA World Cup, found that they have increased their running distance at low intensity. Activities of lower intensity, such as jogging and walking, tend to dominate players' work-rate profiles, emphasizing the predominantly aerobic nature of the game (Faude et al., 2012; Chmura et al., 2018). However, several authors have stressed the importance of sprints and very high-intensity running for the match outcomes (Andrzejewski et al., 2018; Chmura et al., 2018). Therefore, despite increasing fatigue, it was important to maintain and even improve high intensity distance covered and the number of sprints, especially during the knockout stage. Results of Rey et al. (2010) prove that players who played two matches in the week after the previous game, perform less sprints and feature an overall reduction in the length of distance covered at a high intensity. The distances covered by players and the intensity of their physical work are well known to change during the match (Mohr et al., 2003, Aquino et al., 2020), which, when considered in isolation, could suggest that either accumulative fatigue (driving toward total physiological system failure) is a feature of elite match-play or that there is a defined pacing strategy in place (to defend a regulated level of exercise homeostasis) (Edwards, 2009). It was observed in our study that the MEP can employ pacing strategies in consecutive rounds of the World Cup by increasing the distance covered with low intensity to maintain the key activity of high intensity. However, despite the pacing of players, residual fatigue accumulated over successive matches and subsequent incomplete recovery can have the effect of decreasing physical performance, especially in MEP who play in most matches (Nédélec et al., 2013; Arruda et al., 2015; Silva et al., 2017; Chmura et al., 2019). This is especially noticeable in the knockout phase after the rotation in the third match, where both the distance covered at low intensity and the distance covered at high intensity show increasing trends. Loch et al. (2019) suggests that during this time in MEP the use of individualized methods of regeneration should be applied allowing effective reduction of residual fatigue and accelerated regeneration. Our analysis showed that another way to complement the pacing strategy may be to introduce squad rotation, which in the context of residual fatigue may reduce the risk of a decline in high-intensity physical activity parameters.

## Limitations and Strengths

The authors are fully aware of many factors that could have influenced the results of the presented analyzes. Players who take part in the final tournament of the World Cup need to be adequately prepared physically, mentally, as well as technically and tactically (Drust et al., 2012). Therefore, our analyzes allow us to examine only part of the factors influencing the decisions made by coaches during the tournament. It would be important to use analyses of, among other things, exercise tests. Unfortunately, these data are proprietary. The analysis should also include other variables such as injuries or residual fatigue trips during one tournament. Further research using kinematic data of several consecutive elite players matches is needed.

The strength of this study is that, for the first time, all 4 teams that made it to the seventh game of the tournament rotate MEP the most in the third game of the group phase. The analysis takes into account variables such as the number of minutes played and matches during a single tournament. In this way it is possible to determine the most exploited players during the tournament and to study how their bodies react to the progressive residual fatigue. Additionally, the analysis examines MEP changes of pacing of physical activity by introducing an extended rest period between the second and fourth matches in consecutive rounds of World Cup.

## Conclusion

Practitioners and coaches should be more aware what possibilities are offered by squad rotation. It allows coaches to manipulate the rest period of the most exploited players and check the form of rested players. The squad rotations applied in the third match of the group phase allowed the coaches to prepare the most exploited players for the more important matches, minimizing the accumulation of fatigue before the knockout phase, where any defeat means the end of the tournament. This information could be beneficial to coaches regarding optimizing the match running performances of their players during the international tournament.

From a practical point of view, squad rotation can be a valuable support to a pacing strategy. Analysis of the top 4 teams at the World Cup in Russia confirms that coaches used the third game of the group phase and introducing rested players who played less in another tournament matches. It has been also shown that MEPs in the knockout stage use pacing strategies to maintain high intensity parameters by increasing the proportion of low-intensity runs. This means that in congested schedules MEPs are able to control their activity despite increasing residual fatigue. Thus, they reduce overload and the risk of injury.

## Data Availability Statement

The raw data supporting the conclusions of this article will be made available by the authors, without undue reservation.

## Ethics Statement

The studies involving human participants were reviewed and approved by Senate Ethics Committee in University School of Physical Education in Wrocław. The ethics committee waived the requirement of written informed consent for participation.

## Author Contributions

PC, MKoł, MA, JC, and MKon: conceptualization and writing—review and editing. PC, MKoł, and MKon: methodology. MKon: software, formal analysis, resources, and data curation. MKoł and AR: validation and visualization. PC and MKon: investigation. PC, MKoł, and MA: writing—original draft preparation. MKoł and MA: supervision. PC: project administration. AR and JC: funding acquisition. All authors contributed to the article and approved the submitted version.

## Conflict of Interest

The authors declare that the research was conducted in the absence of any commercial or financial relationships that could be construed as a potential conflict of interest.

## Publisher's Note

All claims expressed in this article are solely those of the authors and do not necessarily represent those of their affiliated organizations, or those of the publisher, the editors and the reviewers. Any product that may be evaluated in this article, or claim that may be made by its manufacturer, is not guaranteed or endorsed by the publisher.
